# Utility of iliac crest tetracycline-labelled bone biopsy in osteoporosis and metabolic bone disease: An evaluation of 95 cases over a period of 25 years

**DOI:** 10.1016/j.bonr.2023.101715

**Published:** 2023-09-24

**Authors:** Juan M. Colazo, Julia Quirion, Anthony D. Judice, Jennifer Halpern, Herbert S. Schwartz, S. Bobo Tanner, Joshua M. Lawrenz, Kathryn M. Dahir, Ginger E. Holt

**Affiliations:** a Medical Scientist Training Program, Vanderbilt University School of Medicine, Nashville, TN, USA; b Division of Musculoskeletal Oncology, Department of Orthopaedic Surgery, Vanderbilt University Medical Center, Nashville, TN, USA; c Department of Medicine, Division of Rheumatology and Immunology, Vanderbilt University Medical Center, Nashville, TN, USA; d Department of Medicine, Division of Endocrinology and Diabetes, Vanderbilt University Medical Center, Nashville, TN, USA

**Keywords:** Metabolic bone disease, Osteoporosis, Osteomalacia, Bone biopsy, Antibiotic-labelled bone biopsy, Tetracycline labelling

## Abstract

**Background:**

Metabolic bone diseases (MBD) are typically diagnosed by non-invasive imaging and clinical biomarkers. However, imaging does not provide structural information, and biomarkers can be transiently affected by many systemic factors. Bone biopsy and pathologic evaluation is the gold standard for diagnosis of MBD, however, it is rarely utilized. We describe our technique for iliac crest tetracycline-labelled bone using a cannulated drill and assess the utility of bone biopsies to provide diagnostic and therapeutic guidance.

**Methods:**

In the 25-year period between March 1998 and January 2023, a total of 95 bone biopsies were performed on 94 patients for an osteological indication at Vanderbilt University Medical Center (VUMC). Patient demographics, bone biopsy indications, complications, diagnostic utility, and subsequent therapeutic guidance were retrospectively reviewed and analyzed.

**Results:**

The procedure had minimal complications and was well tolerated by patients. This technique provided good quality specimens for pathology, which helped establish a diagnosis and treatment change in most patients. Patients that had biopsy-guided treatment alterations showed significant increases in Dual-Energy X-ray Absorptiometry (DEXA) bone mineral density (BMD) scores post-biopsy and subsequent treatment.

**Conclusion:**

Despite scientific and technological progress in non-invasive diagnostic imaging, clinical biomarkers, and procedures for MBD, there remains a small but significant subset of patients who may benefit from inclusion of tetracycline-labelled bone biopsy into the diagnostic and therapeutic picture. Future prospective comparison studies are warranted.

**Mini abstract:**

Tetracycline-labelled bone biopsies are under-utilized. Biopsy led to a histological diagnosis and ensuing treatment alteration in most patients with significant increases in bone mineral density. The biopsy procedure used herein provided good specimens with low pain/adverse events. Bone biopsy remains a valuable tool in a small, though significant, subset of patients.

## Highlights

• Antibiotic-labelled bone biopsies are under-utilized in metabolic bone disease.
• A novel bone biopsy procedure with less pain/adverse events is described herein.
• A histological diagnosis and ensuing treatment alteration was seen in most patients.
• Increases in bone mineral density were seen after biopsy-guided treatment alterations.
• Bone biopsy is a valuable tool in a small, though significant, subset of patients.

## 1 Introduction

Metabolic bone disease (MBD) is an entity that encompasses a wide array of primary and secondary disorders that affect the overall quality of bone. There are multiple pathologic processes that affect bone quality that can be found in both pediatric and adult patient populations. The most well-known entity in this regard is osteoporosis which affects an estimated 10 million people older than 50 in the United States alone (Sozen et al., 2017). Chronic kidney disease-mineral and bone disorders (CKD-MBD) have recently been adopted to describe many of these pathologic processes (Carvalho et al., 2016).

Bone mineral density (BMD) is measured by absorption of X-ray energy (DXA), a method which is only able to quantify the physical interaction between X-ray energy and calcium-containing crystalline structures under strict conditions (Writing Group for the IPDC, 2004). Although an important clinical advancement, DXA is unable to distinguish between different metabolic bone diseases and histopathological abnormalities influencing X-ray absorption. Confounding variables that affect DXA interpretation include patient positioning/movement, scoliosis, enthesis calcification, degenerative joint changes, hardware, and other artifacts (Martineau et al., 2021). HRpQCT and Trabecular Bone Score images (from DXA) and bone micro-indentation are new technologies that provide some structural information and can enhance fracture risk prediction but, unlike bone biopsies, they do not provide information on bone turnover status and thus cannot indicate what treatment may be most suitable for the patient (Popp et al., 2014; Ramalho et al., 2018; Nyman et al., 2016). Measurement of BMD and Trabecular Bone Score (TBS) by DXA has shown to predict fracture risk and treatment response in statistical analyses of large cohorts (Cranney et al., 2002). However, DXA cannot diagnose specific conditions in individual patients, especially in atypical situations (Greene et al., 2009; Li et al., 2010; Rojo Venegas et al., 2012). Osteomalacia, one form of MBD, occurs as a result of insufficient mineralization, and can have normal DXA scans (Bhan et al., 2018). In addition to DXA, many of the clinical laboratory biomarkers for bone turnover and remodeling (CTX, NTX, PINP, PICP, ALP, bone ALP, OC, etc. (Greenblatt et al., 2017)) lack sensitivity and specificity and may vary based upon fasting/non fasting, diurnal patterns, age/sex, pregnancy, and medications, thus painting only part of the systemic osteological picture (Martineau et al., 2021; Sprague et al., 2016).

One important step in diagnosing MBD is differentiating between calcified and uncalcified bone matrix. Tetracycline double labelled bone biopsy dates back at least to 1969 (Frost et al., 1969; Frost, 1969; Wu and Frost, 1969) and takes advantage of the property of this class of antibiotics to be deposited along the front of active calcification in bone and therefore allows visualization of active bone formation under fluorescent microscopy. Patients are given tetracycline or declomycin prior to bone biopsy to allow for dynamic analysis of osteoblastic/osteoclastic activity and mineralization processes, which can help distinguish low turnover from high turnover disease (Dalle Carbonare et al., 2021; Dalle Carbonare and Giannini, 2004). Bone biopsy allows for evaluation of histomorphometric patterns, which vary significantly between MBD's and can therefore assist in diagnosis. Bone biopsy is indicated to further clarify cases of unexplained fragility fractures or to evaluate the effects of different treatments on bone (Dempster et al., 2001; Dempster et al., 2016a; Dempster et al., 2016b), such as antiresorptive or anabolic drugs and their potential side effects (Odvina et al., 2005). The study of localization, levels of expression, and synthesis of important targets in bone and its microenvironment is now possible through application of in situ hybridization histochemistry (ISHH) and/or immunohistochemistry (IHC). ISHH allows study of specific mRNA expression and IHC determines the presence and distribution of target protein in cells. Combining the established bone histomorphometric techniques with ISHH and IHC has the ability to elevate the diagnostic and therapeutic utility of this procedure (Langub et al., 2000).

The iliac crest bone biopsy has long been the gold standard for diagnosis of MBD however, it is a rather uncommonly performed procedure as it requires coordination with a surgeon well versed in the technique and needs of the requesting service (Carvalho et al., 2016; Malluche et al., 2007). At Vanderbilt University Medical Center (VUMC), the orthopaedic oncology department has collaborated with the endocrinology, rheumatology, and nephrology departments to perform multiple bone biopsies every year. Our team believes this is an underutilized tool not only for diagnosis but for evaluation of response to treatment or accumulation of disease burden over time. For example, bone biopsies can be used at baseline and follow up in disorders of osteomalacia where DXA scan is an inadequate surrogate marker (Kim et al., 1988). Furthermore, DXA normative values are inappropriate for comparison of BMD for patients with rare skeletal dysplasias.

In this series, we describe the VUMC method of iliac crest tetracycline-labelled biopsy and the diagnostic/therapeutic utility of the procedure in a cohort of 94 patients (95 biopsies) across 25 years.

## 2 Methods

### 2.1 Retrospective chart review

This study was approved by the Institutional Review Board of Vanderbilt University Medical Center. In the period between March 1998 and January 2023, a total of 95 bone biopsies on 94 patients were performed for an osteological indication. Retrospective chart review was conducted by a single adjudicator to identify the medical history as well as the bone-biopsy guided treatment and post-biopsy procedure adverse events (comparing “traditional” technique vs. “new” technique described below). Study data was collected and managed using Research Electronic Data Capture (REDCap) electronic data capture tools hosted at VUMC.

### 2.2 Bone biopsy technique

**Fig. 1:**
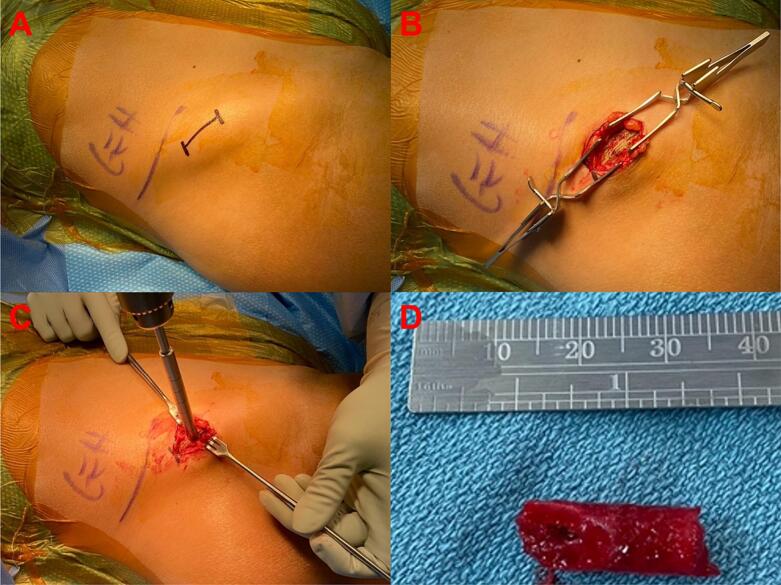
Iliac Crest Tetracycline-labelled Biopsy Procedure (Post-2018). A) Patient positioned supine, prepped and draped with the iliac crest marked (2 cm incision). B) The iliac crest is exposed with subcutaneous tissues retracted, the periosteum of the ilium is identified and peeled off the crest medially and laterally. C) A guide pin is positioned between the inner and outer table of the ilium and advanced until cortical bone is felt distally. The Arthrex™ collared pin is then inserted into the guide pin hole and the Arthrex™ 8 mm diameter cannulated coring reamer is advanced over the collared pin. D) Final sample containing inner cortical bone, cancellous bone, outer cortical bone, and superior cortical bone (tri-cortical specimen).

There have been technique papers published regarding the procedure for obtaining trans-iliac bone (Bordier et al., 1964; Faugere and Malluche, 1983; Jamshidi et al., 1971). Prior to biopsy, the patient was prescribed and completed a course of Declomycin to label the trabecular bone. Before 2018, under general anesthesia a 5–7 cm incision was made on the iliac crest and dissection carried through the gluteus muscles to the periosteum. Some surgeons used a saw to cut a wedge of bone from the iliac crest. Others used the Anspach™ (DePuy Synthes, Johnson & Johnson, Raynham, MA, USA) with a cutting cone cylindrical burr make a bicortical pass through the ilium approximately 2 cm posterior and 2 cm inferior to the anterior superior iliac spine. In 2018, the biopsy technique was altered. Prior to biopsy the patient is prescribed and completes a course of antibiotic therapy to label the trabecular bone. Declomycin 300 mg 3 times daily (TID) is given on days 1 and 2 and then day 13, 14, 15, 16 with outpatient surgery being performed on day 17. Biopsy is performed in the operating room under general anesthesia. In the operating room, the patient is positioned supine, and the iliac crest is sterilely prepped into the field (Fig. 1A). A horizontal 2 cm incision is carried down through skin and subcutaneous tissue just superior to the anterior superior iliac spine. The periosteum of the ilium is identified and peeled off the crest medially and laterally (Fig. 1B). A guide pin is positioned between the inner and outer table of the ilium and advanced until cortical bone is felt distally. The Arthrex® (Naples, FL, USA) collared pin is then inserted into the guide pin hole and the Arthrex® 8 mm diameter cannulated coring reamer (AR-8901CR) is advanced over the collared pin (Fig. 1C). The reamer is then advanced along the pin through the near cortex until the far cortex is encountered. The reamer is then toggled in the channel to remove the core of bone as a single cylinder for better pathologic analysis of overall architecture (Fig. 1D). The wound is then irrigated with 50 mL of warm saline and Surgicel® (Ethicon, Johnson & Johnson, Cincinnati, OH, USA) and Gelfoam® (Pfizer, New York City, NY, USA) is placed into the defect until hemostasis is achieved. Approximately 10 cc's of 0.5 % Marcaine with Epinephrine diluted 1:200,000 is administered into the incisional soft tissues before closure and application of a sterile dressing. After biopsy, patients are discharged home with no weightbearing restrictions, and are seen at two weeks postoperatively for follow up and removal of sutures.

### 2.3 Histological preparation

The core specimen is put in 70 % ethanol upon collection and shipped in 70 % ethanol. The antibiotic-labels are light sensitive and therefore samples should be kept in the dark. Samples are sent to the University of Kentucky Division of Nephrology, Bone and Mineral Metabolism where for processing and analysis of biopsies, which includes modified Masson-Goldner stain, aluminum stains (aurintricarboxylic acid and acid solochrome azurine), amyloid deposit stain (Congo red) and iron stain (Gomori). Unstained slides are also prepared for fluorescent, polarized light, and phase contrast microscopy. Processing of the bone samples are performed without decalcification as described previously (Hahn et al., 1991). Histopathological diagnosis is made by an experienced osteopathologist. Approximately 4–5 weeks are required to process the specimen and generate results.

### 2.4 Statistical analyses

Only patients that had both pre-biopsy and post-biopsy scores were included in the statistical analysis. Pre-biopsy DXA corresponds to the closest scan to biopsy date, while post-biopsy DXA is most recent imaging, providing the longest follow up. Patients with pre-biopsy T-scores or *Z*-scores >2 were removed (Osteopetrosis). Statistical analysis was done by paired two-tailed *t*-test with *p*-values shown above graphs and lines connecting points are one specific patient pre- and post-biopsy. All statistical analysis was performed with GraphPad Prism Version 9.5.0 (525), November 8, 2022 (GraphPad, Dotmatics, Boston, MA, USA).

## 3 Results

The average age was 49 (±14) years, and the median age was 50 years with a range from 22 to 80 years (Table 1). Out of 94 patients, 39 (42 %) were male and 55 (58 %) were female of which 23 (42 %) were pre-menopausal and 32 (58 %) were post-menopausal. Out of 94 patients, 75 were White, 17 were Black, and 2 were Hispanic or Latino. 36 patients had End-stage renal disease (ESRD). Interestingly 100 % of the Black patients in this study had ESRD and 14/17 (82 %) were referred for bone biopsy to determine parathyroidectomy candidacy meanwhile only 3/17 (18 %) were referred for diagnostic/treatment purposes. This is in contrast with White patients in which 18/75 (24 %) had ESRD, 14/75 (19 %) were referred for parathyroidectomy candidacy and 61/75 (81 %) were referred for diagnostic/treatment purposes.

**Table 1:** Patient cohort demographics.

Total Patients, n (%)	94
Patients with > 1 Biopsy	1 (1.1)
Age	
Median (Range)	50 (22–80)
Age, Mean (+/-SD)	48.99 (±13.47)
– <30 years	8 (8.5 %)
– 30–40 years	19 (20.2 %)
– 40–50 years	17 (18.1 %)
– 50–60 years	28 (29.8 %)
– >60 years	22 (23.4 %)
Gender, n (%)	
Male	39 (41.5)
Female	55 (58.5)
– Pre-menopausal	23/55 (41.8)
– Post-menopausal	32/55 (58.2)
Ethnicity, n (%)	
White	75 (79.8)
Black	17 (18.1)
Hispanic or Latino	2 (2.1)
End-Stage Renal Disease (ESRD)	36 (38.3)
– White	18 (24)
– Black	17 (100)
– Hispanic or Latino	1 (50)
Parathyroidectomy Candidacy Bone Biopsy Indication	29 (30.8)
– White	14 (18.7)
– Black	14 (82.4)
– Hispanic or Latino	1 (50)
All Other Bone Biopsy Indications	65 (69.1)
– White	61 (81.3)
– Black	3 (17.6)
– Hispanic or Latino	1 (50)

The most common indications for bone biopsy were osteoporosis in a male or pre-menopausal female without a known underlying cause. Other indications included parathyroidectomy candidacy, decreasing BMD or fracture continuation despite osteoporotic therapy, differentiating between osteoporosis and osteomalacia, unable to tolerate current medications, patients with atraumatic fractures with normal DXA BMD, and others (Table 2). Out of 94 patients, only one patient had a second biopsy to determine therapeutic improvement with the second biopsy occurring concurrently with total knee replacement, a case report previously published (Colazo et al., 2020).

**Table 2:** Biopsy indications.

Indications for bone biopsy (May be more than one per patient), n (%)	95
Osteoporotic Male without Endocrine Abnormality (May also have another indication)	39 (41.1)
Parathyroidectomy Candidacy	29 (30.5)
Osteoporotic Pre-menopausal Female without Endocrine Abnormality (May also have another indication)	23 (24.2)
Decreasing BMD or fractures despite current or previous anti-osteoporotic treatment	20 (21.1)
Further Diagnostics i.e., atypical fractures, determine turnover for therapy guidance, rule out Hypophosphatasia	12 (12.6)
To diagnose Osteoporosis (potentially renal) vs Osteomalacia for potential treatment	7 (7.4)
Side effects/unable to tolerate current/previous treatment	4 (4.2)
Fractures despite low-to-normal bone density	4 (4.2)
Abnormal Lab Values Follow up	3 (3.2)
Diagnosed with Hypophosphatasia, turnover status if patient should remain on Teriparatide (Asfotase alfa not approved at the time)	1 (1.1)
Postpartum/lactation related osteoporosis (PLO)	1(1.1)
To determine treatment response (Only patient with 2 bone biopsies)	1 (1.1)

**Fig. 2:**
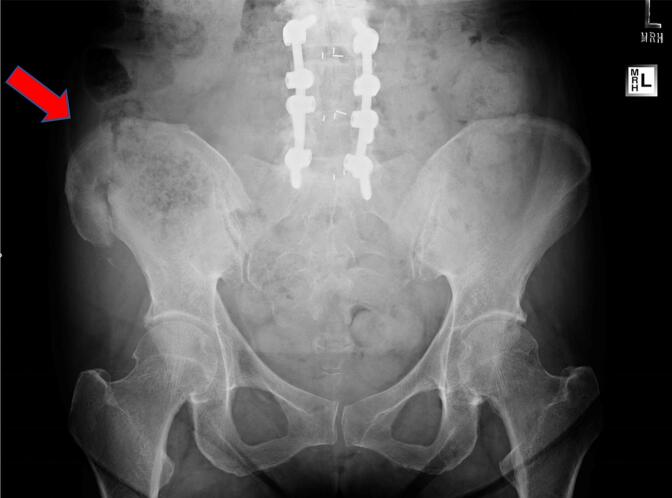
Fracture at the Iliac-crest Biopsy Site in a Patient in the Pre-2018 Technique Group (Red Arrow).

The mean sample length was 1.03 cm (SD = 0.31 cm) and the mean sample diameter was 1.18 cm (SD = 0.49 cm). Out of 95 bone biopsies, 89 (94 %) had post-biopsy follow-up; 18/89 (20 %) with the post-2018 technique and 71/89 (80 %) with the pre-2018 technique. Both procedures had a similar percentage of patients describing mild postoperative pain (1–3/10) (23 % vs. 28 %). However, moderate (3–6/10) or severe (>6/10) postoperative pain was only experienced in the pre-2018 group (Table 3). Furthermore, there were 4 hematomas, 4 patients with transient neuropathy, 1 seroma, and 1 fracture at the bone biopsy site in the pre-2018 technique group with none of these adverse events in the post-2018 technique group. (Table 3). Fracture at the biopsy site can be a catastrophic event especially in these patients where bone quality is already poor. The patient was a Hypophosphatasia (HPP) patient before treatment; the fracture occurred when they were getting out of bed about two weeks after the procedure (Fig. 2). The patient had significant pain and a functional decline requiring crutches and wheelchair for mobilization. In this patient, the information gathered from bone biopsy (Anabolic Therapy Recommendation) lead to remaining on treatment course as they were prescribed Teriparatide a month before biopsy (Before HPP Enzyme Replacement Therapy Asfotase Alfa was FDA-approved). Previously, they were treated with anti-resorptive therapy for years with no improvement in DXA BMD and continuous atraumatic fractures.

**Table 3:** Biopsy Adverse Events – Pre-2018 vs. Post-2018 Technique.

Documented Biopsy Procedure Follow-up, n (%) 89/95 (93.7)	Pre-2018 (71/89)	Post-2018 (18/89)
Pain 1–3 (/10) (Greater than normal post-operative pain/discomfort)	16 (22.5)	5 (27.8)
Pain 3–6 (/10) (Greater than normal post-operative pain/discomfort)	10 (14.1)	0
Pain 6–10 (/10) (Greater than normal post-operative pain/discomfort)	3 (4.2)	0
Transient Neuropathy	4 (5.6)	0
Hematoma	4 (5.6)	0
Allergic reactions, gastrointestinal disturbances, and photosensitivity secondary to declomycin intake	2 (2.8)	1 (5.6)
Seroma	1 (1.4)	0
Skin Site Infection (Cellulitis)	1 (1.4)	1 (5.6)
Fracture (Biopsy Site)	1 (1.4)	0

The most common histological diagnoses were Low Turnover Osteoporosis, Renal Osteodystrophy with Secondary Hyperparathyroid Bone Disease, High Turnover Osteoporosis, and Fracturing Renal Osteodystrophy with only one biopsy interpreted as normal bone (Table 4). In general, Low Turnover Osteoporosis and Fracturing Renal Osteodystrophy led to a treatment recommendation of anabolic therapy i.e., teriparatide, abaloparatide, or romosozumab. Meanwhile, High Turnover Osteoporosis often led to a treatment recommendation of anti-resorptive therapy i.e., bisphosphonates or denosumab (RANKL inhibitor). Renal osteodystrophy patients were either recommended parathyroidectomy (if bone biopsy was done for parathyroidectomy candidacy) or treatment with a lowered dose of anti-resorptive therapy.

**Table 4:** Histological diagnosis.

Histological Diagnosis, n (%)	95
Low Turnover Osteoporosis	35 (36.8)
Renal Osteodystrophy with Secondary Hyperparathyroid Bone Disease	25 (26.3)
High Turnover Osteoporosis	13 (13.7)
No Report Available	8 (8.4)
Fracturing Renal Osteodystrophy	6 (6.3)
Consistent with Hypophosphatasia	2 (2.1)
Osteomalacia with trabecular osteopenia	1 (1.1)
Anaplastic large cell lymphoma	1 (1.1)
High turnover osteomalacia	1 (1.1)
Neoplasm causing osteomalacia	1 (1.1)
For Treatment Response	1 (1.1)
Normal Bone Histology	1 (1.1)

Out of 95 bone biopsies, 70 (74 %) led to a treatment alteration with 7 (7 %) leading to confirmation of the current treatment course (Table 5). For bone biopsies done for other reasons than determining parathyroidectomy candidacy, 49/66 (74 %) led to a treatment alteration with 7 (11 %) leading to confirmation of the current treatment (Table 5). Thus, 56/66 (85 %) patients had a treatment recommendation based on their histopathologic analyses. For those that did not have a treatment alteration or confirmation to continue the current treatment course, the most common causes were patient status decline, patient already completing 2 years of teriparatide (maximum time recommended), the treatment not being studied/recommended with the patient's characteristics i.e., transplant, metastatic breast cancer, and the patient rejecting treatment (Table 5).

**Table 5:** Clinical/treatment alterations guided by biopsy results.

Was there a major clinical/treatment alteration due to bone biopsy? (Removing Parathyroidectomy Candidacy), n (%)	66
No	9 (13.6)
Yes	49 (74.2)
Keep Treatment Course	7 (10.6)
Unknown (No Follow Up)	1 (1.5)
Those with No, why? n (%)	9
Patient Status Declined	5 (55.5)
Normal Bone Histology	1 (11.1)
Teriparatide already used for 2 years, romosozumab not studied in transplant patients	1 (11.1)
Teriparatide recommended, but patient history of metastatic breast cancer and prior radiation treatment and therefore unknown risks	1 (11.1)
Patient did not want medication/treatment offered	1 (11.1)
Those with Yes or Keep Treatment Course (55), Did treatment align with histology recommendation? n (%)	55
No	6 (10.9)
Yes	45 (81.8)
Anabolic therapy recommended, patient already completed 2 years of teriparatide treatment, IV zoledronic acid started instead	1 (1.8)
Antiresorptive therapy recommended, patient was already started on teriparatide, and patient was fracturing on actonel therefore teriparatide treatment kept	1 (1.8)
Anabolic therapy recommended, teriparatide only option at the time but patient did not want daily injections, therefore IV pamidronate started for 2 years with no improvement, patient eventually started and completed 2 years of teriparatide	1 (1.8)
No Treatment, but further diagnostics	2 (3.6)
Was there a major treatment alteration due to bone biopsy? (Including Parathyroidectomy Candidacy), n (%)	95
No	10 (10.5)
Yes	70 (73.7)
Keep Treatment Course	7 (7.4)
Unknown (No Follow Up)	8 (8.4)
Those with No, why? n (%)	10
Patient Status Decline	6 (60)
Normal Bone Histology	1 (10)
Teriparatide already used for 2 years, romosozumab not studied in transplant patients	1 (10)
Teriparatide recommended, but patient history of metastatic breast cancer and prior radiation treatment and therefore unknown risks	1 (10)
Patient did not want medication/treatment offered	1 (10)
Those with Yes or Keep Treatment Course (76), Did treatment align with histology recommendation? n (%)	76
No	6 (7.9)
Yes	66 (86.8)
Anabolic therapy recommended, patient already completed 2 years of teriparatide treatment, IV zoledronic acid started instead	1 (1.3)
Antiresorptive therapy recommended, patient was already started on teriparatide, and patient was fracturing on actonel, therefore teriparatide treatment kept	1 (1.3)
Anabolic therapy recommended, teriparatide only option at the time but patient did not want daily injections, therefore IV pamidronate started for 2 years with no improvement, patient eventually completed 2 years of teriparatide	1 (1.3)
No Treatment, but further diagnostics	2 (2.6)

The treatment alterations due to biopsy guidance included medication addition(s), parathyroidectomy surgery, genetic testing/consult, further diagnostics, removal of medication and addition of medications, Vanderbilt Undiagnosed Disease Network (UDN) enrollment, recruitment to clinical trials, or an attempt to correct electrolytes (Table 6). The 3 most common medications prescribed were Teriparatide (Anabolic Therapy), Zoledronic acid (Anti-resorptive), and Denosumab (Anti-resorptive). Seven patients were directed to stop a current medication to start the histology-guided medication although many patients had stopped their medications after referral to Vanderbilt but pre-biopsy referral due to side effects, no BMD improvement, and/or recurrent fractures despite the treatment. Thus, one can speculate that many patients may be put on an initial treatment that does not fit their pathologic profile.

**Table 6:** Treatment alteration types guided by bone biopsy results.

Major Treatment Alteration Types in Patients (May be More than One Per Patient), n (%)	68
Addition of Medication(s)	38 (55.9)
Parathyroidectomy Surgery (For Renal Osteodystrophy with Hyperparathyroid Bone Disease)	22 (32.4)
Genetic Testing/Consult	9 (13.2)
Further Diagnostics	8 (11.8)
Removal of Medication(s) and Addition of Medication(s)	7 (10.3)
Added to VUMC Undiagnosed Disease Network	3 (4.4)
Recruited to Clinical Trial	1 (1.5)
Attempt to Correct Electrolyte Balances i.e., Magnesium	1 (1.5)

Biopsy-directed Medication Additions (Throughout Follow-Up), FDA Approval Year	
Teriparatide, 2002	31
Zoledronic Acid, 2001	10
Denosumab, 2010	8
Alendronate, 1995	4
Pamidronate, 1991	4
Calcitonin, 1991	1
Romosozumab, 2019	3
Abaloparatide, 2017	3
Hormone Replacement Therapy, 1998	2
Risedronate, 1998	1
Asfotase Alfa, 2015	1
Calcitriol, 1978	1

**Fig. 3:**
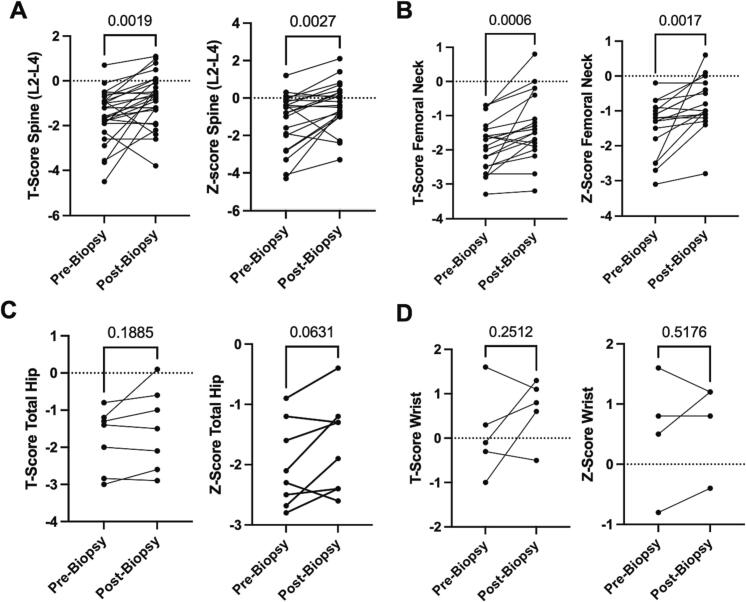
DXA Bone Mineral Densities (BMD) Pre-Biopsy and Post-Biopsy Display Potential Biopsy-guided Improvements. A) Pre-biopsy and post-biopsy T-score of the spine (L2-L4) (*n* = 27) and Pre-biopsy and post-biopsy *Z*-score of the spine (L2-L4) (*n* = 22). B) Pre-biopsy and post-biopsy T-score of the femoral neck (*n* = 21) and Pre-biopsy and post-biopsy *Z*-score of the femoral neck (18). C) Pre-biopsy and post-biopsy T-score of the total hip (*n* = 7) and Pre-biopsy and post-biopsy Z-score of the total hip (*n* = 8). D) Pre-biopsy and post-biopsy T-score of the wrist (*n* = 5) and Pre-biopsy and post-biopsy Z-score of the wrist (*n* = 4). Only patients that had pre-biopsy and post-biopsy scores included. Pre-biopsy DXA scan is closest scan to biopsy date, post-biopsy DXA scan is most recent (longer follow-up); patients with T-scores or Z-scores >2 were removed. Statistical analyses were done by paired *t*-test with *p*-values shown above graphs and lines connecting points are one specific patient pre- and post-biopsy.

Pre-biopsy and post-biopsy/treatment alteration DXA T-scores and *Z*-scores in multiple anatomical locations were collected and statistically analyzed using a paired *t*-test. From an individual basis, there was a significant increase in BMD in the spine (L2-L4), the femoral neck, and the total hip with an insignificant difference in wrist BMD (Fig. 3).

## 4 Discussion

Prevalence rates of osteoporosis and osteopenia are higher in women than in men, and, in both genders, the rate of osteoporosis increases significantly with age. Using the World Health Organization (WHO) criteria, 30 % of White postmenopausal women in the US have osteoporosis, and 54 % have osteopenia (De Martinis et al., 2021). For these reasons, it's not surprising that 66 % of patients (62/94) in the current study referred for bone biopsy were either males or pre-menopausal females and this trend was also seen in other bone biopsy cohort studies (Kann et al., 2006). Although a large proportion of the cases were to determine aluminum-negative and hyperparathyroid bone disease for parathyroidectomy candidacy, these were mostly all performed from 1998 to 2004. From 2004 to 2023, ESRD patients were referred much less frequency, and if so, not for surgical candidacy, but to provide medication recommendations. The majority of referred patients from 2004 to 2023 were patients with either undiagnosed causes of MBD or failed osteoporotic treatment. Interestingly, 100 % of Black patients referred for bone biopsy had ESRD, and the majority (82 %) were referred only for parathyroidectomy candidacy. Meanwhile, White patients were much less likely to carry an ESRD diagnosis and were much more likely to receive a bone biopsy for bone-related workup and treatment. This coincides with multiple studies describing disparities in bone health (Hamrick et al., 2006).

Osteoporosis treatment usually starts with an antiresorptive agent and switches to an anabolic agent if it fails. It has been shown that over-suppressing bone resorption may result in reduced bone formation and increased fracture risk (Bauer et al., 2004) and that patients with prior treatment with antiresorptive agents may have reduced response to subsequent anabolic treatment (Ettinger et al., 2004; Miller et al., 2008). Therefore, initially prescribing the appropriate treatment type for the patient's bone turnover state is paramount and could significantly change osteoporosis and MBD outcomes (Malluche et al., 2022). With an ever-increasing landscape of therapeutic options for MBD, direct histological visualization may allow for more individualized treatment recommendations and response (Rojo Venegas et al., 2012; Mori and Zhou, 2016). Paired bone biopsies i.e., one before treatment and one after treatment, which are performed in clinical trials, are the gold standard, but very few patients will consent to this because of the procedure (Dempster et al., 2001; Takahata et al., 2022; Chavassieux et al., 2014; Miller and McCarthy, 2015). Repeat biopsies to guide therapy are underutilized in clinical practice as evident by only one patient in our study. For these reasons, techniques using a smaller incision with less complications are imperative.

When comparing the pre-2018 technique with the post-2018 technique, patients were less likely to experience moderate/severe pain with reduced complications. Furthermore, since adapting this procedure protocol at VUMC, we have yet to observe a fracture through the biopsy site, a potential catastrophic event for this patient cohort. We believe implementation of this technique will provide better patient outcomes and may allow for a higher percentage of patients providing consent for biopsies.

In this study, pathological analysis of bone through bone biopsy allowed for a definitive histological diagnosis not provided by imaging or lab studies. Of importance, only one bone biopsy in this cohort was interpreted as histological normal bone (compared to 8/99 in the study by Kann et al. (Kann et al., 2006)). Most patients had major treatment alterations as a direct result of the bone biopsy and most aligned with the histological therapy recommendation. Although retrospective in nature, we were able to quantify changes in DXA T-scores and *Z*-scores pre-biopsy and post-biopsy with subsequent treatment alteration, observing a significant overall increase in BMD. These results show that bone biopsy is a useful diagnostic tool that can lead to a definitive diagnosis which guides treatment, to the overall benefit of the patient.

This study is limited by its retrospective nature, although 95 is a substantial number for this rarely performed procedure. Given that these cases were collected over the course of 25 years, certain FDA-approved therapeutics were not available at the time of biopsy/treatment initiation. For example, RANKL inhibitor denosumab (2010), sclerostin-inhibitor romosozumab (2019), asfotase Alfa (2017), and abalaparotide (2017) are four therapies prescribed in this study that were only recently approved, so many patients and prescribing physicians in this study did not have these options available. Teriparatide was the only anabolic therapy available throughout portions of this study and therefore may be over-represented in this cohort compared to a more modern cohort. In some analyses, there was no information due to loss of follow up. For pre-biopsy and post-biopsy DXA scans, there were no defined time intervals as would be in a prospective study. Doses and length of treatment were not documented and therefore pharmacokinetic and pharmacodynamic effects cannot be inferred or determined. For these reasons, future prospective studies are warranted where one could consider performing routine DXA scan follow-ups and comparisons to non-invasive metabolic bone diagnostic tests, imaging, and/or biomarkers.

## 5 Conclusion

Iliac crest tetracycline-labelled biopsy is a valuable tool in diagnosing MBD and guiding treatment. The technique used in this study yields useful samples with minimal postoperative pain and complications. Overall, these results support the use of biopsies in a carefully selected subset of patients and warrant future prospective studies.

## Funding statement

JMC is supported by NIGMS of the 10.13039/100000002National Institutes of Health under award number T32GM007347. The content in this report is solely the responsibility of the authors and does not necessarily represent the official views of the National Institutes of Health.

## CRediT authorship contribution statement

**Juan M. Colazo:** Writing – review & editing, Writing – original draft, Visualization, Validation, Resources, Project administration, Methodology, Investigation, Formal analysis, Data curation, Conceptualization. **Julia Quirion:** Writing – review & editing, Supervision, Investigation. **Anthony D. Judice:** Formal analysis, Data curation, Conceptualization. **Jennifer Halpern:** Formal analysis, Data curation. **Herbert S. Schwartz:** Writing – review & editing, Supervision, Investigation, Formal analysis, Data curation. **S. Bobo Tanner:** Writing – review & editing, Supervision, Investigation, Formal analysis, Data curation. **Joshua M. Lawrenz:** Writing – review & editing, Writing – original draft, Supervision, Methodology, Investigation, Formal analysis, Data curation, Conceptualization. **Kathryn M. Dahir:** Supervision, Methodology, Formal analysis, Data curation, Conceptualization. **Ginger E. Holt:** Writing – review & editing, Writing – original draft, Validation, Supervision, Methodology, Investigation, Funding acquisition, Formal analysis, Data curation, Conceptualization.

## Declaration of competing interest

Juan M. Colazo, Julia Quirion, Anthony D. Judice, Jennifer Halpern, Herbert S. Schwartz, S. Bobo Tanner, Kathryn M. Dahir, Joshua M. Lawrenz, and Ginger E. Holt declare that they have no conflict of interest.

## Data Availability

Information and data can be accessed from the corresponding authors.
